# Hamate Fracture

**Published:** 2015-06-04

**Authors:** T. Snoap, J. Habeck, T. Ruiter

**Affiliations:** ^1^Department of Orthopedic Surgery, Western Michigan University Homer Stryker MD School of Medicine; ^2^Borgess Medical Center, Kalamazoo, MI

**Keywords:** hamate fracture, carpal fracture, carpus, body of hamate, open reduction internal fixation

**Figure:**
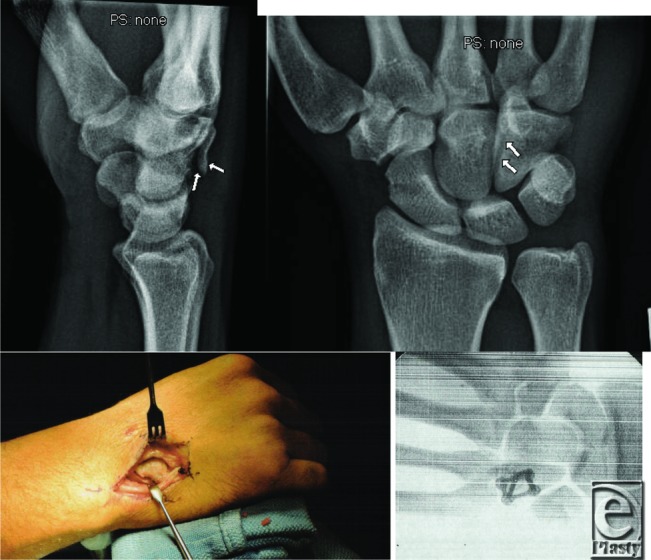


## DESCRIPTION

A 39-year-old mechanic presented with a 6-week history of activity-related ulnar sided wrist pain in his dominant right hand after punching injury. History, physical examination, and imaging were consistent with a hamate body fracture nonunion. The patient underwent open reduction internal fixation and went on to bony union and restoration of function.

## QUESTIONS

1. **How are hamate fractures classified?**
2. **What is the mechanism of injury of hamate fractures?**
3. **How are hamate fractures diagnosed?**
4. **What is the treatment of hamate fractures?**

## DISCUSSION

Fractures of the carpal bones with the exclusion of the scaphoid are rare entities, accounting for approximately 1.1% of all fractures.^1^ Of the carpal bones, the hamate accounts for only 2% of fractures as compared with the scaphoid, which accounts for 70%.^2^^,^^3^ Hamate fractures can be classified broadly on the basis of the Milch classification into fractures of either the hook or the body.

The mechanism of injury to the hamate dictates the fracture type and orientation. Fractures of the hook are commonly seen in golfers, baseball players, and racket sport players. Falls and crush injuries are also common injury mechanisms. In comparison, body fractures of the hamate are more associated with the mechanism of a clenched fist striking a wall.^4^

Hamate fractures can pose a diagnostic challenge for the treating physician. The rarity of the injury as well as the complex anatomy of the carpal bones can make this diagnosis commonly missed. In the Ebraheim et al^5^ small case series of coronal body fractures of the hamate, the time from injury to definitive diagnosis ranged from 2 days to 5 weeks, with the average being 10 days. Patients commonly describe pain at the hypothenar eminence that is aggravated via direct palpation or gripping. The hook of the hamate pull test is a dynamic test commonly used to assess for hook fractures. Resisted flexion of the fourth and fifth digits displaces the fracture and causes pain.^2^ Because of the hamate's intimate location with the ulnar nerve, patients can present with symptoms ranging from paresthesias to weakness. Imaging can assist with diagnosis of these injuries. The overlap of the hook of the hamate on the body can lead to difficulty picking up these fractures on standard hand series radiographs. Often a carpal tunnel view or supinated oblique view can better identify the fracture. Computed tomographic scan can aid in diagnosis and assist in preoperative planning.

The treatment of hamate fractures varies on the basis of displacement and fracture location. For hamate hook fractures that are nondisplaced, short arm cast immobilization can be implemented. Close clinical follow-up is required as the nonunion rate approaches 50%.^6^ Hook fractures that are displaced, or associated with nerve or tendon irritation, can be excised acutely. Evidence has shown that excision does not adversely affect grip strength or wrist range of motion.^7^ This option is technically easier than attempting open reduction internal fixation. Hamate body fractures are managed in a slightly different manner compared with hook fractures. Nondisplaced body fractures are treated in a short arm cast for 4 to 6 weeks until radiographic evidence of union. Displaced fractures require an anatomic reduction to prevent abnormal joint mechanics and soft-tissue irritation. In conjunction with performing open reduction and internal fixation with either compression screws or low-profile plates, these injuries may require temporary fixation across the Carpometacarpal joints to aid in stability. In a study of hamate body fractures in the coronal plane, Wharton et al^8^ reported that using Kirschner wires for displaced fractures often resulted in incomplete or malreduction at follow-up and this correlated to a poor clinical outcome. Rigid fixation is advocated for these fracture patterns but may not always lead to excellent patient outcomes.

In conclusion, hamate fractures are rare entities that can cause significant patient morbidity if not recognized and treated appropriately. Nondisplaced fractures can generally be treated with immobilization and close follow-up. Displaced hook of the hamate fractures can be treated with fragment excision, whereas displaced body fractures generally require open reduction and rigid internal fixation.
